# Intolerance of uncertainty as a prospective predictor of generalized anxiety and depression in adolescents: evidence from a three-wave, 12-month study

**DOI:** 10.1017/S204579602610064X

**Published:** 2026-04-21

**Authors:** Haoxian Ye, Linmao Zou, Zixuan Shao, Jiaqi Wang, Jiaxiong Zhang, Keying Liu, Yiming Zhang, Ranran Wang, Fang Fan

**Affiliations:** 1 School of Psychology, Centre for Studies of Psychological Applications, Guangdong Key Laboratory of Mental Health and Cognitive Science, Guangdong Emergency Response Technology Research Center for Psychological Assistance in Emergencies, South China Normal University, Guangzhou, China; 2 Key Laboratory of Brain, Cognition and Education Sciences (South China Normal University), Philosophy and Social Science Laboratory of Reading and Development in Children and Adolescents (South China Normal University), Ministry of Education, Guangzhou, China; 3 Centre for Teacher Development in Gaozhou, Maoming, China

**Keywords:** adolescents, depression, generalized anxiety, intolerance of uncertainty, predictive ability

## Abstract

**Aims:**

Intolerance of uncertainty (IU) – a dispositional inability to react effectively to uncertain situations – has been increasingly conceptualized as a transdiagnostic risk factor for internalizing problems such as generalized anxiety and depression. However, evidence for its temporal role in the development of these conditions remains limited, particularly in adolescents, a group at heightened risk for psychopathology.

**Methods:**

A total of 5,291 adolescents (46.2% boys; M age = 14.40 ± 1.56, range = 10–18 years) completed self-report measures of IU, generalized anxiety and depressive symptoms at baseline, 6 months and 12 months. Linear and logistic regression analyses examined whether baseline IU predicted subsequent symptom severity and elevated (above-cut-off) symptom levels over time.

**Results:**

Higher baseline IU significantly predicted increases in generalized anxiety and depressive symptoms, as well as higher odds of elevated generalized anxiety and depressive symptom levels at both 6- and 12-month follow-ups, even after adjusting for baseline symptom severity or baseline elevated symptom status. Baseline IU also predicted the new-onset and persistence of elevated symptoms across both intervals. Stratified analyses revealed developmental and sex differences: IU’s predictive effects were strongest in early adolescence for girls and in middle-to-late adolescence for boys.

**Conclusions:**

IU emerged as a transdiagnostic longitudinal predictor of generalized anxiety and depressive symptoms in adolescents, supporting its value as an early screening marker of vulnerability. Interventions targeting IU may offer an effective strategy for reducing broad internalizing risk during this critical developmental period.

## Introduction

Adolescence is a critical developmental stage characterized by rapid biological, psychological and social changes, and it represents a peak period for the onset of mental health disorders (Copeland *et al.*, 2009). Among these, generalized anxiety and depression are especially prevalent, often impairing academic, social and family functioning (Kieling *et al.*, 2024). Their high comorbidity rates further complicate clinical outcomes, leading to more severe, chronic courses and poorer treatment responses compared with either condition alone (Garber and Weersing, 2010). This overlap has motivated researchers to move beyond disorder-specific frameworks towards transdiagnostic mechanisms – the shared processes that contribute to the development and maintenance of internalizing problems.

One of the transdiagnostic factors that has received considerable attention in recent years is intolerance of uncertainty (IU), defined as a dispositional incapacity to tolerate the aversive responses triggered by uncertainty (Carleton, 2016). Individuals high in IU tend to interpret uncertain situations as threatening and anticipate negative personal or behavioural consequences (Carleton, 2016). This cognitive bias undermines effective functioning in ambiguous contexts and gives rise to maladaptive coping responses – such as excessive worry, reassurance seeking, avoidance and compulsive information gathering – that may maintain or exacerbate emotional distress (Bottesi *et al.*, 2019). Recent meta-analytic and empirical evidence has documented robust associations between IU and both generalized anxiety and depression in adolescents (Osmanağaoğlu *et al.*, 2018; Bottesi *et al.*, 2022; Zemestani *et al.*, 2022; Ye *et al.*, 2023; Mares and Fuller, 2026), highlighting its potential as a transdiagnostic construct.

From a risk factor perspective, a construct must demonstrate correlation with the outcome, temporal precedence and malleability to qualify as a true risk factor for psychopathology (Kraemer *et al.*, 2005). Extending this framework, a transdiagnostic risk factor should additionally show predictive effects across multiple forms of psychopathology rather than being confined to a single disorder. Existing research has established the strong concurrent associations between IU and adolescent generalized anxiety and depression (Osmanağaoğlu *et al.*, 2018; Ye *et al.*, 2023; Mares and Fuller, 2026) and has demonstrated that IU can be modified through targeted interventions, including IU-focused cognitive bias modification (Oglesby *et al.*, 2017) and psycho-education (Shapiro *et al.*, 2023). In contrast, evidence establishing its temporal precedence remains limited. Although one longitudinal study suggested that IU may function as an essential prerequisite process for the emergence of generalized anxiety symptoms in adolescents (Marchetti *et al.*, 2025), it remains unclear whether IU prospectively predicts the development of both generalized anxiety and depression, thereby operating as a truly transdiagnostic risk factor rather than a process specific to anxiety or a correlate of concurrent distress. Given that generalized anxiety and depression often first emerge during adolescence (Merikangas *et al.*, 2010) and frequently persist into adulthood (Lewinsohn *et al.*, 1999), establishing the temporal and transdiagnostic predictive role of IU in adolescents may therefore provide a stronger empirical foundation for early preventive interventions targeting transdiagnostic mechanisms of internalizing psychopathology.

Notably, several developmental studies have suggested that adolescents’ cognitive capacities for managing uncertainty emerge and improve as they develop (Roebers *et al.*, 2007; Lyons and Ghetti, 2011), which suggests that both IU levels and their effects on psychopathology may vary across early, middle and late adolescence. Additionally, considering adolescent girls tend to have stronger tendencies towards maladaptive cognitive-emotional styles (e.g., worry and rumination) (Hankin, 2009), the effects of IU on psychopathology may be amplified for girls compared to boys, consistent with evidence of higher rates of generalized anxiety and depression among girls (Allgood-Merten *et al.*, 1990; Lewinsohn *et al.*, 1998). Taken together, these considerations highlight the importance of examining whether IU’s predictive value differs by age stage and sex, which has implications for identifying high-risk subgroups and tailoring preventive efforts.

To address these gaps, this study investigated the role of IU in predicting generalized anxiety and depression in adolescents, using a longitudinal design with two follow-up windows (6 months and 1 year). Considering that generalized anxiety and depression can be conceptualized both dimensionally (i.e., symptom severity on a continuum) and categorically for screening purposes (i.e., above- vs. below-cut-off symptom levels), we examined both types of outcomes to capture variations in symptom severity and elevated symptom status. Specifically, we tested whether baseline IU predicted (1) changes in the severity of generalized anxiety and depressive symptoms; (2) the likelihood of elevated (i.e., above-cut-off) generalized anxiety and depressive symptoms; and (3) transitions in elevated symptom status over time (i.e., new-onset and persistence). Based on prior theory and empirical findings (Osmanağaoğlu *et al.*, 2018; McEvoy *et al.*, 2019), we hypothesized that higher baseline IU would predict greater symptom severity and a higher likelihood of elevated symptom levels (as well as their new-onset and persistence), even after accounting for baseline symptom severity or baseline elevated symptom status. Given limited evidence on age- and sex-specific effects, stratified analyses were conducted exploratorily without specific hypotheses.

## Methods

### Participants

A three-wave survey was conducted among students in five randomly selected primary, middle and high schools in an eastern city of Guangdong, China. Data were collected at 6-month intervals: T1 (March 2024), T2 (September 2024) and T3 (March 2025). The study adhered to the principles of the Helsinki Declaration (2013) and was approved by the Ethics Committee of the corresponding author’s university (SCNU-PSY-2024-119). To ensure data quality, responses were excluded if participants (1) provided incorrect identity information (e.g., invalid student IDs), (2) failed attention-check items (e.g., instructions requiring a specific response) or (3) completed the survey in an unreasonably short time (e.g., <1 s per item). After exclusions, 9,068 adolescents provided valid data at T1, 7,352 at T2 and 5,291 at T3. The final longitudinal sample (*n* = 5,291) had a mean age of 14.40 years (SD = 1.56; range 10–18), with 2,846 girls (53.8%). Age distribution was: early adolescence (10–12 years; n = 830, 15.7%), middle adolescence (13–15 years; *n* = 2,879, 54.4%) and late adolescence (16–18 years; *n* = 1,582, 29.9%). Detailed socio-demographic characteristics are reported in Table S1.

Attrition analyses were conducted to assess potential bias due to student absence, transfer or dropout. Independent *t*-tests and chi-square tests revealed no significant differences between participants who completed all three surveys and those who only participated at T1 in baseline anxiety symptom severity (M = 3.40 vs. 3.41, *p* = 0.949), baseline depressive symptoms severity (M = 4.84 vs. 4.89, *p* = 0.666) or socio-demographic variables (*p*s > 0.05). The Missing Completely at Random test (Little and Rubin, 1989) also indicated non-significant results (*p* = 0.457), suggesting that the pattern of the missing data was not materially different from a random pattern (Bollen, 1989).

### Procedures

Data collection took place during regular school hours in computer rooms, supported by the local education bureau and school mental health departments. Participation was voluntary and confidential, with data used solely for research purposes. Students accessed the web-based survey using anonymized IDs and provided electronic informed consent alongside their caregivers before participation. They were reminded that they could withdraw at any time. To safeguard student well-being, school psychology teachers received crisis intervention training to identify and support at-risk students after each wave of survey. Additionally, a free psychological distress hotline was made available to students and caregivers.

### Measures

#### Intolerance of uncertainty

IU was measured using the Chinese version of the 12-item Intolerance of Uncertainty Scale for Children (Cornacchio *et al.*, 2018). Items are rated on a 5-point Likert scale (1 = not at all to 5 = very much), with higher scores reflecting greater IU. The Chinese version has demonstrated good psychometric properties in Chinese adolescents (Ye *et al.*, 2024). Cronbach’s *α* value was 0.947 at T1.

#### Generalized anxiety

Generalized anxiety symptoms over the past 2 weeks were assessed using the Chinese version of the 7-item Generalized Anxiety Disorder Scale (GAD-7; (Spitzer *et al.*, 2006)). Items are rated on a 4-point scale (0 = not at all to 3 = nearly every day), yielding scores from 0 to 21. A cut-off of 10 indicates elevated generalized anxiety symptoms (Löwe *et al.*, 2008). The Chinese version of the GAD-7 has shown good reliability in Chinese adolescents (Sun *et al.*, 2021). Cronbach’s *α* values were 0.941, 0.945 and 0.954 at T1, T2 and T3, respectively.

#### Depression

Depressive symptoms over the past 2 weeks were measured with the Chinese version of the 9-item Patient Health Questionnaire (PHQ-9; [Kroenke *et al.*, 2001]). Items are rated on the same 4-point scale as the GAD-7, with scores ranging from 0 to 27. A cut-off of 10 indicates elevated depressive symptoms (Manea *et al.*, 2012). The Chinese version of the PHQ-9 has demonstrated reliability in Chinese adolescents (Leung *et al.*, 2020; Gao and Liu, 2024). Cronbach’s *α* values were 0.919, 0.927 and 0.936 at T1, T2 and T3, respectively.

#### Socio-demographic variables

Socio-demographic variables collected at T1 were included as covariates: age stage (early, middle or late adolescence), sex (male/female), single child status (yes/no), left-behind child status (yes/no), immigrants’ child status (yes/no), family history of mental disorders (yes/no), self history of mental disorders (yes/no), self history of chronic physical illness (yes^1^/no), parental marital status (married vs. non-married^2^), parental job stability (yes/no) and parental education (junior high school or below/senior high school/college and above).

### Statistical analyses

First, descriptive statistics summarized sample characteristics, with means (SD) for continuous variables and frequencies for categorical variables. Correlations were also computed to examine preliminary associations among study variables. Besides, since all data were collected through self-report, Harman’s one-factor test was used to assess common method variance. The confirmatory factor analysis (CFA) was also used to analyse the longitudinal measurement invariance of generalized anxiety and depressive symptoms separately to determine whether they exhibited configural, metric and scalar invariance in Mplus 7.4. Given that chi-square values are sensitive to large samples, changes in the comparative fit index (CFI) (ΔCFI ≤ 0.01), root mean square error of approximation (RMSEA) (ΔRMSEA ≤ 0.015) and standardized root mean square residual (SRMR) (metric invariance: ΔSRMR ≤ 0.03; scalar invariance: ΔSRMR ≤ 0.01) were considered indicative of invariant measurement (Chen, 2007).

Then, hierarchical linear regression models tested whether baseline IU predicted changes in generalized anxiety and depression symptom severity at 6-month (T1→T2) and 12-month (T1→T3) follow-ups. Baseline IU, generalized anxiety symptom severity and depression symptom severity were standardized when entered into the models. Unstandardized regression coefficients (*B*), standardized coefficients (*β*), 95% confidence intervals (CIs) and model *R*^2^ values were reported to facilitate interpretation of effect sizes. To complement the dimensional analyses, hierarchical logistic regression models were used to examine whether baseline IU predicted elevated generalized anxiety and depressive symptom levels at two follow-ups. Elevated symptom status was operationalized as scoring at or above established screening cut-off scores on the GAD-7 (≥10) and PHQ-9 (≥10). These cut-off scores used in this study were derived from prior psychometric validation studies of the Chinese versions of these instruments (He *et al.*, 2010; Wang *et al.*, 2014), which have been widely adopted in epidemiological research involving Chinese adolescents (Zhou *et al.*, 2020; Zhang *et al.*, 2021; Wang *et al.*, 2022; Fan *et al.*, 2024). Nonetheless, as optimal cut-off scores may vary slightly across developmental stages, findings based on categorical classifications should be interpreted with caution. Consistent with prior epidemiological research (Li *et al.*, 2021; Wang *et al.*, 2022; Zhang *et al.*, 2023), we also examined whether baseline IU predicted transitions in elevated symptom status. Four trajectories were defined: (1) persistence (above cut-off at both time points), (2) resistance (below cut-off at both time points), (3) new-onset (below cut-off at baseline and above cut-off at follow-up) and (4) remission (above cut-off at baseline and below cut-off at follow-up). Baseline IU was standardized when entered into the models. Odds ratios (ORs), 95% CIs and Nagelkerke pseudo-*R*^2^ values were reported. In all the hierarchical modelling procedures, covariates were entered in sequential steps, with the specific covariate structure varying slightly depending on the outcome examined. Detailed model specifications are provided in the corresponding table notes.

Finally, to explore the potential moderating effect of age stage and sex, stratified linear and logistic regressions were conducted by age stage (early, middle and late adolescence) and sex (boys and girls). Separate analyses were also performed within each sex across age stages to identify sensitive developmental periods for boys and girls.

All analyses were conducted using SPSS 23.0. All tests were two-tailed, with *p* < 0.05 considered statistically significant.

## Results

### Preliminary analyses

Because all data were collected through self-report, Harman’s one-factor test was used to assess common method variance. The first principal factor accounted for 35.6% of the variance, below the 40% threshold, suggesting that common method bias was not a serious concern (Johnson *et al.*, 2011). In addition, CFA results confirmed longitudinal measurement invariance for generalized anxiety and depressive symptoms across the three time points (ΔCFI < 0.01, ΔRMSEA < 0.015, ΔSRMR ≤ 0.01), supporting the comparability of these constructs over time (see Table S5).

Descriptive statistics and correlations among study variables are reported in Table 1. All variables showed acceptable distributions, with absolute skewness values below 2 and kurtosis values below 7 (Kim, 2013). As expected, generalized anxiety and depressive symptoms were strongly correlated both cross-sectionally and longitudinally. The correlations between baseline IU and generalized anxiety and depressive symptoms decreased slightly over time but remained substantial.

**Table 1.** Descriptive statistics and correlation matrix among study variables

Variables	*M*	SD	Skewness	Kurtosis	1	2	3	4	5	6	7
IU level_T1	30.58	10.89	−0.18	−0.60	-						
Generalized anxiety symptom severity_T1	3.40	4.21	1.58	2.83	0.56***	-					
Generalized anxiety symptom severity_T2	3.20	4.06	1.50	2.46	0.38***	0.56***	-				
Generalized anxiety symptom severity_T3	2.83	3.87	1.65	3.26	0.35***	0.50***	0.57***	-			
Depressive symptom severity_T1	4.84	4.92	1.26	1.99	0.55***	0.85***	0.54***	0.49***	-		
Depressive symptom severity_T2	4.60	4.85	1.24	1.83	0.39***	0.55***	0.86***	0.55***	0.58***	-	
Depressive symptom severity_T3	4.08	4.61	1.34	2.38	0.36***	0.48***	0.55***	0.87***	0.50***	0.58***	-

IU: intolerance of uncertainty; *M*: mean; SD: standard deviation.

T1: time 1; T2: time 2; T3: time 3.

* *p*< 0.05, **p< 0.01, ***p< 0.001.

### Effect of IU on symptom severity over time

As shown in Table 2, after adjusting for covariates and baseline symptom severity, higher baseline IU significantly predicted increases in the severity of generalized anxiety (*β* = 0.06 over 6 months; *β* = 0.08 over 12 months) and depressive symptoms (*β* = 0.06 over 6 months; *β* = 0.08 over 12 months). For both generalized anxiety and depressive symptoms, the predictive effect was slightly stronger over the longer interval.

**Table 2.** Linear regressions for the effect of baseline IU on the follow-up severity of generalized anxiety and depressive symptoms

**Predictor (T1)**	**Generalized anxiety symptom severity (T2)**							
	Model 1^a^ (*R*^2^ = 0.143)		Model 2^b^ (*R*^2^ = 0.334)		Model 3^c^ (*R*^2^ = 0.308)		Model 4^d^ (*R*^2^ = 0.343)	
	*B* (95% CI)	*β*	*B* (95% CI)	*β*	*B* (95% CI)	*β*	*B* (95% CI)	*β*
IU level	0.38*** (0.35, 0.40)	0.38	0.07*** (0.04, 0.10)	0.07	0.10*** (0.08, 0.13)	0.10	0.06***(0.03, 0.08)	0.06
**Predictor (T1)**	**Generalized anxiety symptom severity (T3)**							
	Model 1^a^ (R^2^ = 0.126)		Model 2^b^ (R^2^ = 0.263)		Model 3^c^ (R^2^ = 0.252)		Model 4^d^ (R^2^ = 0.272)	
	*B* (95% CI)	*β*	*B* (95% CI)	*β*	*B* (95% CI)	*β*	*B* (95% CI)	*β*
IU level	0.35*** (0.33, 0.38)	0.35	0.10*** (0.07, 0.12)	0.10	0.11*** (0.09, 0.14)	0.11	0.08*** (0.05, 0.11)	0.08
**Predictor (T1)**	**Depressive symptom severity (T2)**							
	Model 1^a^ (R^2^ = 0.149)		Model 2^c^ (R^2^ = 0.351)		Model 3^b^ (R^2^ = 0.326)		Model 4^d^ (R^2^ = 0.360)	
	*B* (95% CI)	*β*	*B* (95% CI)	*β*	*B* (95% CI)	*β*	*B* (95% CI)	*β*
IU level	0.39*** (0.36, 0.41)	0.39	0.08*** (0.06, 0.11)	0.08	0.09*** (0.07, 0.12)	0.09	0.06*** (0.03, 0.09)	0.06
**Predictor (T1)**	**Depressive symptom severity (T3)**							
	Model 1^a^ (R^2^ = 0.126)		Model 2^c^ (R^2^ = 0.267)		Model 3^b^ (R^2^ = 0.249)		Model 4^d^ (R^2^ = 0.273)	
	*B* (95% CI)	*β*	*B* (95% CI)	*β*	*B* (95% CI)	*β*	*B* (95% CI)	*β*
IU level	0.36*** (0.33, 0.38)	0.36	0.10*** (0.07, 0.13)	0.10	0.11*** (0.08, 0.14)	0.11	0.08*** (0.05, 0.11)	0.08

IU: Intolerance of uncertainty; *B*: unstandardized regression coefficient; *β*: standardized regression coefficient; CI: confidence interval.

T1: time 1; T2: time 2; T3: time 3.

a No adjustment.

b Adjusting for socio-demographic variables, and baseline generalized anxiety symptom severity.

c Adjusting for socio-demographic variables, and baseline depressive symptom severity.

d Adjusting for socio-demographic variables, baseline generalized anxiety symptom severity, and baseline depressive symptom severity.

* *p*< 0.05, **p< 0.01, ***p< 0.001.

### Effect of IU on elevated symptom levels over time

As shown in Table 3, baseline IU significantly predicted the likelihood of elevated (above-cut-off) symptom levels at follow-ups. The adjusted odds of elevated generalized anxiety symptoms were 1.56 over 6 months and 1.62 over 12 months. For elevated depressive symptoms, the adjusted odds were 1.55 and 1.53, respectively. These findings indicate that IU’s predictive association with elevated generalized anxiety symptoms strengthened modestly over time, whereas its association with elevated depressive symptoms remained relatively stable. Across both time frames, IU was a slightly stronger predictor of elevated generalized anxiety symptoms than of elevated depressive symptoms.

**Table 3.** Logistic regressions for the effect of baseline IU on the follow-up status of elevated generalized anxiety and depressive symptoms

**Predictor (T1)**	**Elevated generalized anxiety symptoms (T2)**			
	Model 1^a^ (*R*^2^ = 0.113)	Model 2^b^ (*R*^2^ = 0.225)	Model 3^c^ (*R*^2^ = 0.226)	Model 4^d^ (*R*^2^ = 0.251)
	OR (95% CI)	OR (95% CI)	OR (95% CI)	OR (95% CI)
IU level	2.57*** (2.26, 2.92)	1.69*** (1.47, 1.96)	1.74*** (1.51, 2.01)	1.56*** (1.35, 1.80)
**Predictor (T1)**	**Elevated generalized anxiety symptoms (T3)**			
	Model 1^a^ (*R*^2^ = 0.111)	Model 2^b^ (*R*^2^ = 0.212)	Model 3^c^ (*R*^2^ = 0.201)	Model 4^d^ (*R*^2^ = 0.227)
	OR (95% CI)	OR (95% CI)	OR (95% CI)	OR (95% CI)
IU level	2.69*** (2.31, 3.12)	1.73*** (1.46, 2.04)	1.84*** (1.56, 2.17)	1.62*** (1.37, 1.91)
**Predictor (T1)**	**Elevated depressive symptoms (T2)**			
	Model 1^a^ (*R*^2^ = 0.118)	Model 2^c^ (*R*^2^ = 0.250)	Model 3^b^ (*R*^2^ = 0.220)	Model 4^d^ (*R*^2^ = 0.264)
	OR (95% CI)	OR (95% CI)	OR (95% CI)	OR (95% CI)
IU level	2.38*** (2.15, 2.64)	1.67*** (1.49, 1.87)	1.72*** (1.54, 1.92)	1.55*** (1.38, 1.74)
**Predictor (T1)**	**Elevated depressive symptoms (T3)**			
	Model 1^a^ (*R*^2^ = 0.098)	Model 2^c^ (*R*^2^ = 0.201)	Model 3^b^ (*R*^2^ = 0.171)	Model 4^d^ (*R*^2^ = 0.208)
	OR (95% CI)	OR (95% CI)	OR (95% CI)	OR (95% CI)
IU level	2.31*** (2.05, 2.59)	1.62*** (1.42, 1.84)	1.69*** (1.49, 1.92)	1.53*** (1.34, 1.74)

IU: intolerance of uncertainty; OR: odds ratio; CI: confidence interval; *R*^2^: Nagelkerke pseudo-*R*.

T1: time 1; T2: time 2; T3: time 3.

a No adjustment.

b Adjusting for socio-demographic variables, and baseline status of elevated generalized anxiety symptoms.

c Adjusting for socio-demographic variables, and baseline status of elevated depressive symptoms.

d Adjusting for socio-demographic variables, baseline status of elevated generalized anxiety symptoms, and baseline status of elevated depressive symptoms.

* *p*< 0.05, **p< 0.01, ***p< 0.001.

### Effect of IU on transitions in elevated symptom status

**Figure 1.**
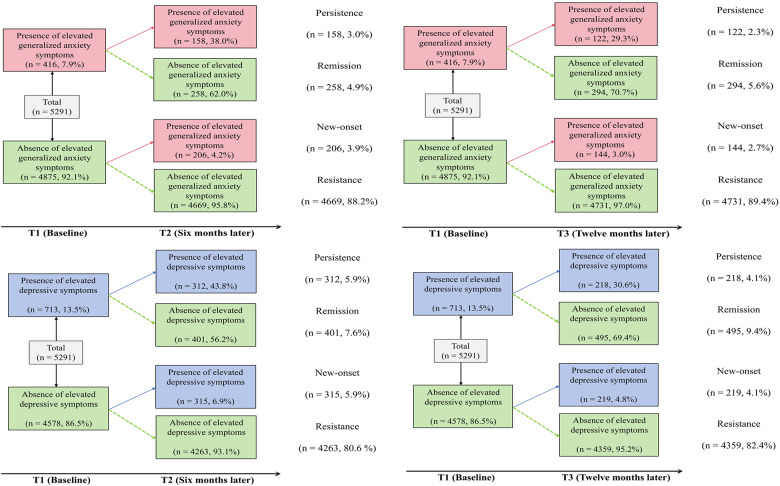
Transitions in status of elevated generalized anxiety and depressive symptoms.

Figure 1 illustrates the four transition patterns, and Table 4 presents the regression models. After adjusting for covariates and baseline elevated symptom status, higher baseline IU significantly predicted the new-onset of elevated generalized anxiety symptoms (OR = 1.61 over 6 months; OR = 1.76 over 12 months) and elevated depressive symptoms (OR = 1.49 over 6 months; OR = 1.71 over 12 months). These findings suggest that IU’s predictive effect on new-onset elevated symptoms strengthened over time. In addition, IU significantly predicted the persistence of elevated generalized anxiety symptoms (OR = 1.68 over 6 months; OR = 1.36 over 12 months) and elevated depressive symptoms (OR = 1.36 over 6 months; OR = 1.24 over 12 months). These results indicate that IU was more strongly associated with persistence in the short term, with attenuated effects over the longer interval. Overall, IU more strongly predicted both new-onset and persistence of elevated generalized anxiety symptoms than of elevated depressive symptoms across both time frames.

**Table 4.** Logistic regression for the effects of baseline IU level on the new-onset and persistence of elevated generalized anxiety and depressive symptoms

**Predictor (T1)**	**Elevated generalized anxiety symptoms**											
	**New-onset vs. Resistance**						**Persistence vs. Remission**					
	**Short-term (from T1 to T2)**			**Long-term (from T1 to T3)**			**Short-term (from T1 to T2)**			**Long-term (from T1 to T3)**		
	Model 1^a^ (*R*^2^ = 0.035)	Model 2^b^ (*R*^2^ = 0.049)	Model 3^c^ (*R*^2^ = 0.085)	Model 1^a^ (*R*^2^ = 0.045)	Model 2^b^ (*R*^2^ = 0.059)	Model 3^c^ (*R*^2^ = 0.076)	Model 1^a^ (*R*^2^ = 0.045)	Model 2^b^ (*R*^2^ = 0.110)	Model 3^c^ (*R*^2^ = 0.135)	Model 1^a^ (*R*^2^ = 0.017)	Model 2^b^ (*R*^2^ = 0.087)	Model 3^c^ (*R*^2^ = 0.106)
	OR (95% CI)	OR (95% CI)	OR (95% CI)	OR (95% CI)	OR (95% CI)	OR (95% CI)	OR (95% CI)	OR (95% CI)	OR (95% CI)	OR (95% CI)	OR (95% CI)	OR (95% CI)
IU level	1.76*** (1.55, 2.01)	1.70***(1.48, 1.94)	1.61*** (1.41, 1.85)	1.96*** (1.62, 2.38)	1.93*** (1.58, 2.35)	1.76*** (1.44, 2.16)	1.70*** (1.28, 2.27)	1.69*** (1.24, 2.30)	1.68*** (1.23, 2.29)	1.39* (1.04, 1.87)	1.37* (1.01, 1.87)	1.36* (1.01, 1.86)
**Predictor (T1)**	**Elevated depressive symptoms**											
	**New-onset vs. Resistance**						**Persistence vs. Remission**					
	**Short-term (from T1 to T2)**			**Long-term (from T1 to T3)**			**Short-term (from T1 to T2)**			**Long-term (from T1 to T3)**		
	Model 1^a^ (*R*^2^ = 0.043)	Model 2^b^ (*R*^2^ = 0.067)	Model 3^d^ (*R*^2^ = 0.077)	Model 1^a^ (*R*^2^ = 0.038)	Model 2^b^ (*R*^2^ = 0.054)	Model 3^d^ (*R*^2^ = 0.055)	Model 1^a^ (*R*^2^ = 0.033)	Model 2^b^ (R^2^ = 0.100)	Model 3^d^ (*R*^2^ = 0.149)	Model 1^a^ (*R*^2^ = 0.024)	Model 2^b^ (*R*^2^ = 0.082)	Model 3^d^ (*R*^2^ = 0.115)
	OR (95% CI)	OR (95% CI)	OR (95% CI)	OR (95% CI)	OR (95% CI)	OR (95% CI)	OR (95% CI)	OR (95% CI)	OR (95% CI)	OR (95% CI)	OR (95% CI)	OR (95% CI)
IU level	1.75*** (1.49, 2.05)	1.68*** (1.43, 1.98)	1.49*** (1.25, 1.76)	1.76*** (1.51, 2.05)	1.74*** (1.48, 2.03)	1.71*** (1.46, 2.01)	1.51*** (1.24,1.85)	1.50*** (1.21, 1.86)	1.36** (1.09, 1.70)	1.45*** (1.17, 1.79)	1.44** (1.15, 1.80)	1.24* (1.01, 1.55)

IU: intolerance of uncertainty; OR: odds ratio; CI: confidence interval; *R*^2^: Nagelkerke pseudo-*R*.

T1: time 1; T2: time 2; T3: time 3.

a No adjustment.

b Adjusting for socio-demographic variables.

c Adjusting for socio-demographic variables, and baseline status of elevated depressive symptoms.

d Adjusting for socio-demographic variables, and baseline status of elevated generalized anxiety symptoms.

* *p*< 0.05, **p< 0.01, ***p< 0.001.

### Stratified analyses by age stage and sex

Stratified analyses (Tables S2–S4) revealed meaningful differences by sex and age stage. Among boys, IU was more strongly associated with symptom severity and elevated symptom levels over 6 months, whereas among girls, these associations were stronger over 12 months. Regarding transitions in elevated symptom status, IU more strongly predicted new-onset elevated symptoms in boys at 6 months and in girls at 12 months. However, IU predicted persistence primarily in the short term for both sexes.

In age-stratified analyses, middle adolescents exhibited the strongest short-term associations between IU and symptom severity, whereas early and late adolescents showed stronger long-term associations. For elevated symptom levels, early adolescents were more vulnerable to elevated depressive symptoms in the short term and to elevated generalized anxiety symptoms in the long term. In contrast, middle and late adolescents consistently showed stronger IU effects on elevated symptom levels in the short and long term, respectively. Regarding transitions, IU predicted new-onset elevated symptoms most strongly for middle adolescents in the short term and for late adolescents in the long term. For early adolescents, IU’s effects on new-onset elevated generalized anxiety symptoms emerged more strongly in the long term, whereas its effects on new-onset elevated depressive symptoms were stronger in the short term. Persistence effects were observed only among middle adolescents and only in the short term.

**Figure 2.**
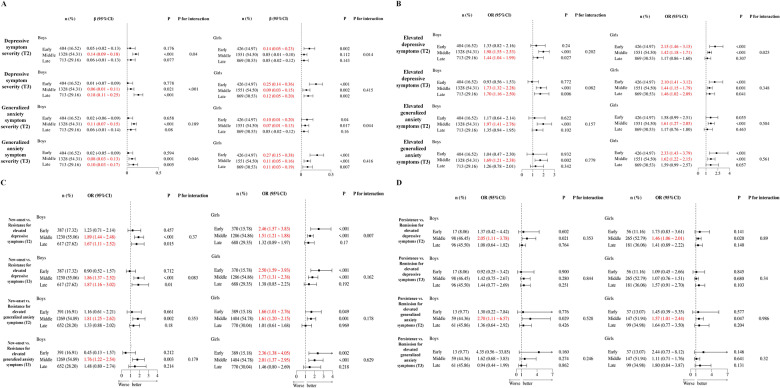
Stratified analyses results. (a) Effect of IU on the follow-up severity of generalized anxiety and depressive symptoms; (b) Effect of IU on the follow-up status of elevated generalized anxiety and depressive symptoms; (c) Effect of IU on the new-onset of elevated generalized anxiety and depressive symptoms; (d) Effect of IU on the persistence of elevated generalized anxiety and depressive symptoms).

Sex-specific analyses across age stages (Fig. 2) provided further detail. For symptom severity (Fig. 2), IU was most strongly associated with increased generalized anxiety and depressive symptoms among girls in early adolescence across both time frames. For boys, the strongest associations appeared in middle adolescence over 6 months and in late adolescence over 12 months. For elevated symptom levels (Fig. 2b–2d), IU predicted new-onset elevated symptoms most strongly among girls in early adolescence and boys in middle-to-late adolescence. Persistence effects again emerged only among middle adolescents, regardless of sex and only in the short term.

## Discussion

To our knowledge, this is the first study to demonstrate the temporal precedence of IU in predicting the development of generalized anxiety and depressive symptoms during adolescence. Our central finding was that baseline IU significantly predicted both subsequent symptom severity and elevated (above-cut-off) symptom levels at 6- and 12-month follow-ups, even after controlling for baseline status. These robust effects extend previous cross-sectional research by showing that IU is not merely a concomitant feature of internalizing problems or a reflection of symptom stability, but an independent and transdiagnostic vulnerability factor in adolescents.

Prior studies have linked IU to subsequent generalized anxiety and depressive symptoms under the extraordinary circumstances of the COVID-19 pandemic (Raymond *et al.*, 2022). Our findings move beyond this unique and highly stressful context, demonstrating IU’s transdiagnostic risk role in more common adolescent growing environments. Moreover, while the predictive power of IU on generalized anxiety and depression has been observed in clinical populations with sufficiently severe psychopathology (Hunt *et al.*, 2022) and in non-clinical adults with mature cognitive systems (Huang *et al.*, 2019; Sahib *et al.*, 2024), this study provides the first evidence that IU prospectively predicts these outcomes in non-clinical adolescents. This expands our understanding of the populations that are affected by IU’s transdiagnostic risks and underscores its importance for etiological models of internalizing psychopathology. Notably, the observed predictive effects of IU were modest in magnitude in our study, which suggested that IU should be viewed as one among several vulnerability mechanisms that collectively contribute to generalized anxiety and depression. Yet, given the high prevalence of internalizing problems during adolescence, even small increases in risk associated with IU may translate into meaningful public health implications at the population level.

While IU showed transdiagnostic risks, its predictive effect was stronger for generalized anxiety than for depression in adolescents, consistent with theoretical models that position IU as central to generalized anxiety rather than depression (Dugas *et al.*, 1998). This pattern likely reflects the more immediate alignment between IU and the cognitive-affective mechanisms of anxiety: individuals with high IU tend to interpret ambiguity as threatening, leading to heightened anticipatory threat appraisal, worry and avoidance (Grupe and Nitschke, 2013). These processes directly mirror the defining features of generalized anxiety, making IU a proximal driver of anxious arousal and vigilance (Dugas and Koerner, 2005). In contrast, IU’s influence on depression may unfold more indirectly and over longer timescales. Persistent difficulty tolerating uncertainty can sustain anxious apprehension and cognitive over-engagement, which, when prolonged and unresolved, may erode one’s sense of control and efficacy, eventually fostering helplessness, hopelessness, anhedonia and withdrawal (Miranda *et al.*, 2008; Yook *et al.*, 2010). Thus, IU may serve as an ‘upstream’ risk factor for depression, operating primarily through sustained anxiety and rumination rather than direct affective reactivity. In this sense, IU functions as a core and proximal mechanism in generalized anxiety but a secondary and distal mechanism in depression, suggesting that it exerts transdiagnostic yet process-specific influences across internalizing conditions. Accordingly, early interventions targeting IU-related anxious processes may yield downstream benefits in preventing depression for adolescents.

Based on the current longitudinal design with two follow-ups, we further found that IU’s transdiagnostic risk effects on symptom severity were slightly stronger at the 12-month follow-up than at 6 months. This result suggests that IU may function as a relatively stable vulnerability factor whose impact accumulates over time. In the short term, symptom stability explains most outcome variance, limiting IU’s additional contribution. Over longer periods, however, the enduring influence of IU on maladaptive coping strategies – such as worry and avoidance – becomes more evident (Yao *et al.*, 2022), thereby fostering symptom severity. Thus, these findings highlight IU’s relevance for shaping the long-term trajectories of adolescent internalizing symptoms and emphasize its value as an early risk marker even when corresponding symptoms are not yet fully expressed.

Our study also revealed nuanced sex- and age-specific patterns. Boys exhibited stronger short-term associations of IU with symptom severity and elevated symptom levels, whereas girls demonstrated stronger long-term effects. Such sex differences may reflect gendered socialization patterns in how adolescents learn to perceive, interpret and cope with uncertainty (Zlomke and Hahn, 2010; Duarte *et al.*, 2015): Girls are typically encouraged to be more emotionally expressive and relationally attuned, but they also tend to internalize stress through worry, rumination and self-focused coping (Nolen-Hoeksema and Girgus, 1994; Zlomke and Hahn, 2010). Such cognitive-emotional styles may amplify the long-term impact of IU, as intolerance towards uncertainty can continuously trigger repetitive negative thinking and sustained emotional arousal. This process may lead to a gradual accumulation of anxiety and depressive symptoms over time. By contrast, boys are more often socialized to suppress or minimize emotional expression and to rely on immediate problem-solving or avoidance-based coping strategies (Tamres *et al.*, 2002; Chaplin and Aldao, 2013). When faced with uncertainty, this coping pattern may result in acute but short-lived distress, consistent with our finding that IU predicted short-term symptom escalation in boys. In addition, age differences also emerged. Middle adolescence emerged as a sensitive window, with IU exerting the strongest short-term influence on both symptom severity and elevated symptom levels, possibly due to heightened emotional reactivity coupled with immature regulation capacities. By contrast, in late adolescence, IU’s impact grew more pronounced over time, indicating that improved regulatory skills may initially buffer against IU, but cumulative academic, social and identity-related stressors eventually exacerbate its impact. In early adolescence, IU’s risks unfolded differently: while impact on symptom severity emerged more strongly in the long term, impact on elevated symptom levels appeared to emerge in a symptom-shifting trajectory – short-term effects were strongest for depression and long-term effects for anxiety. These above discrepancies may reflect the asynchrony of cognitive-emotional processing across the whole adolescence (Labouvie-Vief, 2015). Notably, IU’s predictive effects were strongest in early adolescence for girls and in middle-to-late adolescence for boys, despite the fact that persistence effects of elevated symptoms emerged only in middle adolescence for both sexes. Taken together, our findings suggest that while IU operates as a transdiagnostic risk across adolescence, its temporal dynamics differ by sex and age stage, which underscores the need for tailored preventive interventions. For boys, interventions should emphasize early identification and short-term support, particularly at middle adolescence when IU’s effects are most acute. For girls, efforts should focus on long-term monitoring and prevention, even in the absence of immediate symptoms, especially during early adolescence when IU confers delayed but enduring risk.

Theoretically, the present findings provided novel insights into the current transdiagnostic perspective proposed in both the Research Domain Criteria (RDoC) and Hierarchical Taxonomy of Psychopathology (HiTOP) frameworks (Dalgleish *et al.*, 2020). Within the RDoC framework, IU maps onto a key cognitive-affective process in the Negative Valence Systems domain, particularly relevant to potential threat and uncertainty processing. Its ability to predict generalized anxiety and depression supports its role as a transdiagnostic mechanism rather than a disorder-specific correlate. Similarly, from the HiTOP perspective, IU may underlie the ‘distress’ spectrum common to internalizing psychopathology like generalized anxiety and depression. By demonstrating IU’s prospective predictive power across different internalizing symptom domains, our study supports the view that shared mechanisms may contribute to the co-occurrence of anxiety and depressive symptoms during adolescence.

Beyond theoretical implications, the present findings have important translational relevance for early prevention and intervention. Given that IU is a valuable early marker for identifying adolescents at elevated future risks for generalized anxiety and depression, incorporating IU assessments into schools and primary care settings could enable earlier identification of high-risk adolescents and timely intervention. Moreover, as baseline IU prospectively predicted the new-onset of elevated generalized anxiety and depressive symptoms among adolescents without elevated baseline symptoms, preventive programmes should directly target IU. At the universal prevention level, school-based curricula could integrate brief modules aimed at enhancing uncertainty tolerance, including mindfulness practices for accepting unpredictability, metacognitive training to challenge intolerance-related beliefs and graded exposure to ambiguous situations. Such approaches align with transdiagnostic cognitive-behavioural therapy (CBT) principles by addressing shared vulnerability processes across emotional disorders and thus offer efficient, scalable prevention strategies. For adolescents already experiencing clinically significant symptoms, evidence-based interventions – such as IU-focused CBT (Dugas and Robichaud, 2007) – that emphasize structured exposure to uncertainty, cognitive restructuring of negative beliefs about unpredictability and behavioural experiments promoting flexible responding, have demonstrated transdiagnostic efficacy in reducing both IU and psychiatric symptoms (Boswell *et al.*, 2013). Accordingly, directly targeting IU in prevention and intervention programmes may help interrupt early risk trajectories and yield broad protective benefits across internalizing psychopathologies.

Despite the valuable insights gained from this study, several limitations should be acknowledged. First, the sample was drawn from a single city, limiting generalizability. Future studies should recruit more diverse populations across regions and backgrounds to enhance external validity. Second, reliance on self-report measures may introduce potential bias. In particular, elevated symptom status was defined using validated screening cut-offs on self-report instruments rather than clinician-administered diagnostic interviews. Therefore, our categorical analyses should be interpreted as reflecting elevated (above-cut-off) symptom levels rather than formal psychiatric diagnoses. Future studies incorporating structured clinical interviews would help validate and extend the present findings. Nonetheless, screening-defined elevated symptom levels have demonstrated predictive utility in identifying adolescents at heightened risk for later clinically significant mental health problems in community settings. Accordingly, our findings should be understood as highlighting IU’s role in forecasting elevated internalizing symptoms rather than confirmed diagnostic onset. Third, although the present longitudinal design with 6- and 12-month intervals allows for preliminary temporal inference, its observational design prevents definitive causal conclusions. Further research should conduct intervention and experimental studies to determine whether reducing IU decreases subsequent increases in generalized anxiety and depressive symptoms. Moreover, considering that the relationship between IU and internalizing symptoms is also possibly bidirectional rather than strictly unidirectional, future research should employ cross-lagged panel models or even dynamic structural equation modelling to more precisely delineate the reciprocal temporal dynamics between IU and internalizing symptoms, clarifying whether IU functions as an antecedent vulnerability, a maintaining factor, or both. Finally, while our focus on non-clinical adolescents is valuable for identifying early risk factors before interventions or medications influence symptom presentation, replication in clinical samples is necessary to establish applicability to treatment contexts.

## Conclusions

In conclusion, this study provides longitudinal evidence that IU is an independent and stable transdiagnostic risk factor for generalized anxiety and depression in adolescents. These findings advance theoretical models of adolescent internalizing psychopathology and highlight IU as a promising early intervention target. Future research should clarify the mechanisms through which IU confers risk and test the efficacy of IU-focused interventions for promoting adolescent mental health.

## Supporting information

10.1017/S204579602610064X.sm001 Ye et al. supplementary material Ye et al. supplementary material

## Data Availability

Data can be made available upon reasonable request. Please contact Fang Fan, PhD, at fangfan@scnu.edu.cn for data supporting the findings of the current study.

## Footnotes

1 Arthritis, angina, asthma, diabetes, visual impairment or hearing problems.

2 Separated/divorced/widowed.

## Supplementary material

The supplementary material for this article can be found at https://doi.org/10.1017/S204579602610064X.
