# Supplementary material for: Intolerance of uncertainty as a prospective predictor of generalized anxiety and depression in adolescents: evidence from a three-wave, 12-month study
Source: Epidemiol Psychiatr Sci. 2026 Apr 21;35:e27. doi: 10.1017/S204579602610064X (PMC13122542; doi:10.1017/S204579602610064X)
Supplement: Ye et al. supplementary material [file S204579602610064Xsup001.docx]

**Table S1** Descriptive statistics of the sample (n = 5,291).

|  | | **n** | **%** |
| --- | --- | --- | --- |
| Age ^a^ [year, mean(SD)] | 14.40 ± 1.56 | - | - |
| Age stage | Early adolescents | 830 | 15.7 |
|  | Middle adolescents | 2879 | 54.4 |
|  | Late adolescents | 1582 | 29.9 |
| Sex | Boys | 2445 | 46.2 |
|  | Girls | 2846 | 53.8 |
| Parental martial status | Married | 4834 | 91.4 |
|  | Non-married ^b^ | 457 | 8.6 |
| Immigrants’ child status | Yes | 462 | 8.7 |
| Single child status | Yes | 246 | 4.6 |
| Left-behind child status ^c^ | Yes | 1047 | 19.8 |
| Father’s education level | Junior high school or less | 3127 | 59.1 |
|  | Senior high school | 1460 | 27.6 |
|  | College or above | 704 | 13.3 |
| Mother’s education level | Junior high school or less | 3719 | 70.3 |
|  | Senior high school | 1031 | 19.5 |
|  | College or above | 541 | 10.2 |
| Father’s job stability | Yes | 4098 | 77.5 |
| Mother’s job stability | Yes | 3898 | 73.7 |
| Family history of mental disorders | Yes | 61 | 1.2 |
| Self history of mental disorders | Yes | 24 | 0.5 |
| Self history of chronic physical illness | Yes | 99 | 1.9 |

**Note**：

^a^ The range of age in the current sample was 10-18 years.

^b^ Non-married included separated, divorced, and widowed.

^c^ Live separately from one or both parents for more than 6 months.

**Table S2** Stratified analyses for the effect of baseline IU on the follow-up severity of generalized anxiety and depressive symptoms.

|  | **Generalized anxiety symptom severity (T2)^#^** | | | **Generalized anxiety symptom severity (T3)^#^** | | | **Depressive symptom severity (T2)^#^** | | | **Depressive symptom severity (T3)^#^** | | |
| --- | --- | --- | --- | --- | --- | --- | --- | --- | --- | --- | --- | --- |
|  | **B (95% CI)** | **β** | **R^2^** | **B (95% CI)** | **β** | **R^2^** | **B (95% CI)** | **β** | **R^2^** | **B (95% CI)** | **β** | **R^2^** |
| Sex | | | | | | | | | | | | |
| Boys | 0.07 (0.04, 0.11)^***^ | 0.07 | 0.279 | 0.06 (0.03, 0.10)^***^ | 0.06 | 0.251 | 0.09 (0.05, 0.12)^***^ | 0.09 | 0.303 | 0.07 (0.03, 0.10)^***^ | 0.07 | 0.241 |
| Girls | 0.05 (0.01, 0.09)^*^ | 0.05 | 0.349 | 0.11 (0.06, 0.15)^***^ | 0.10 | 0.266 | 0.04 (0.01, 0.08)^*^ | 0.03 | 0.359 | 0.11 (0.06, 0.15)^***^ | 0.10 | 0.268 |
| Age stage | | | | | | | | | | | | |
| Early adolescence | 0.06 (-0.01, 0.12) | 0.06 | 0.377 | 0.13 (0.07, 0.20)^***^ | 0.13 | 0.216 | 0.08 (0.02, 0.14)^*^ | 0.07 | 0.373 | 0.12 (0.07, 0.18)^***^ | 0.11 | 0.185 |
| Middle adolescence | 0.08 (0.04, 0.12)^***^ | 0.07 | 0.331 | 0.07 (0.03, 0.11)^***^ | 0.07 | 0.259 | 0.07 (0.04, 0.11)^***^ | 0.06 | 0.363 | 0.06 (0.03, 0.10)^***^ | 0.06 | 0.272 |
| Late adolescence | 0.04 (-0.01, 0.09) | 0.03 | 0.347 | 0.09 (0.04, 0.14)^***^ | 0.09 | 0.310 | 0.04 (-0.01, 0.09) | 0.04 | 0.348 | 0.11 (0.04, 0.18)^***^ | 0.11 | 0.313 |

**Note:**

^#^ Adjusting for sociodemographic variables, baseline generalized anxiety symptom severity, and baseline depressive symptom severity.

^*^p＜0.05, ^**^p＜0.01, ^***^p＜0.001.

T1: time 1; T2: time 2; T3: time 3.

IU: Intolerance of uncertainty; B: unstandardized regression coefficient; β: standardized regression coefficient; CI: confidence interval.

**Table S3** Stratified analyses for the effect of baseline IU on the follow-up status of elevated generalized anxiety and depressive symptoms.

|  | **Elevated generalized anxiety symptoms (T2)^#^** | | **Elevated generalized anxiety symptoms (T3)^#^** | | **Elevated depressive symptoms (T2)^#^** | | **Elevated depressive symptoms (T3)^#^** | |
| --- | --- | --- | --- | --- | --- | --- | --- | --- |
|  | **OR (95% CI)** | **Nagelkerke pseudo-R²** | **OR (95% CI)** | **Nagelkerke pseudo-R²** | **OR (95% CI)** | **Nagelkerke pseudo-R²** | **OR (95% CI)** | **Nagelkerke pseudo-R²** |
| Sex | | | | | | | | |
| Boys | 1.61 (1.29, 2.01)^***^ | 0.202 | 1.47 (1.14, 1.90)^**^ | 0.231 | 1.71 (1.43, 2.04)^***^ | 0.224 | 1.55 (1.27, 1.89)^***^ | 0.177 |
| Girls | 1.50 (1.24, 1.81)^***^ | 0.283 | 1.72 (1.38, 2.14)^***^ | 0.234 | 1.44 (1.24, 1.67)^***^ | 0.271 | 1.53 (1.29, 1.81)^***^ | 0.221 |
| Age stage | | | | | | | | |
| Early adolescence | 1.53 (1.09, 2.15)^*^ | 0.372 | 1.99 (1.35, 2.94)^***^ | 0.302 | 1.89 (1.42, 2.51)^***^ | 0.358 | 1.63 (1.22, 2.17)^***^ | 0.213 |
| Middle adolescence | 1.76 (1.45, 2.13)^***^ | 0.262 | 1.66 (1.34, 2.06)^***^ | 0.201 | 1.64 (1.42, 1.90)^***^ | 0.265 | 1.57 (1.32, 1.86)^***^ | 0.207 |
| Late adolescence | 1.25 (0.95, 1.65) | 0.253 | 1.42 (1.02, 1.98)^*^ | 0.294 | 1.30 (1.04, 1.62)^*^ | 0.258 | 1.56 (1.21, 2.03)^***^ | 0.247 |

**Note:**

^#^ Adjusting for sociodemographic variables, baseline status of elevated generalized anxiety symptoms, and baseline status of elevated depressive symptoms.

^*^p＜0.05, ^**^p＜0.01, ^***^p＜0.001.

T1: time 1; T2: time 2; T3: time 3.

IU: Intolerance of uncertainty; OR: odds ratio; CI: confidence interval.

**Table S4** Stratified analyses for the effects of baseline IU on the new-onset and persistence of elevated generalized anxiety and depressive symptoms in a short term (from T1 to T2) and a long term (from T1 to T3).

|  | **Elevated generalized anxiety symptoms** | | | | | | | |
| --- | --- | --- | --- | --- | --- | --- | --- | --- |
|  | **New-onset vs. Resistance** | | | | **Persistence vs. Remission** | | | |
|  | **Short-term (from T1 to T2)^a^** | | **Long-term (from T1 to T3)^a^** | | **Short-term (from T1 to T2)^a^** | | **Long-term (from T1 to T3)^a^** | |
|  | **OR (95% CI)** | **Nagelkerke pseudo-R²** | **OR (95% CI)** | **Nagelkerke pseudo-R²** | **OR (95% CI)** | **Nagelkerke pseudo-R²** | **OR (95% CI)** | **Nagelkerke pseudo-R²** |
| Sex | | | | | | | | |
| Boys | 1.53 (1.14, 2.06)^**^ | 0.082 | 1.51 (1.18, 1.94)^***^ | 0.099 | 1.85 (1.10, 3.11)^*^ | 0.197 | 1.31 (0.79, 2.17) | 0.320 |
| Girls | 1.47 (1.17, 1.84)^***^ | 0.101 | 1.93 (1.46, 2.56)^***^ | 0.090 | 1.59 (1.11, 2.29)^*^ | 0.203 | 1.36 (0.94, 1.96) | 0.078 |
| Age stage | | | | | | | | |
| Early adolescence | 1.49 (1.01, 2.18)^*^ | 0.200 | 1.85 (1.19, 2.86)^**^ | 0.201 | 1.72 (0.77, 3.85) | 0.231 | 2.75 (0.95, 7.18) | 0.166 |
| Middle adolescence | 1.88 (1.45, 2.45)^***^ | 0.117 | 1.71 (1.37, 2.14)^***^ | 0.088 | 1.77 (1.20, 2.61)^**^ | 0.157 | 1.23 (0.83, 1.82) | 0.096 |
| Late adolescence | 1.17 (0.84, 1.62) | 0.077 | 1.49 (1.01, 2.29)^*^ | 0.109 | 1.48 (0.87, 2.52) | 0.199 | 1.29 (0.76, 2.21) | 0.186 |
|  | **Elevated depressive symptoms** | | | | | | | |
|  | **New-onset vs. Resistance** | | | | **Persistence vs. Remission** | | | |
|  | **Short-term (from T1 to T2)^b^** | | **Long-term (from T1 to T3)^b^** | | **Short-term (from T1 to T2)^b^** | | **Long-term (from T1 to T3)^b^** | |
|  | **OR (95% CI)** | **Nagelkerke pseudo-R²** | **OR (95% CI)** | **Nagelkerke pseudo-R²** | **OR (95% CI)** | **Nagelkerke pseudo-R²** | **OR (95% CI)** | **Nagelkerke pseudo-R²** |
| Sex | | | | | | | | |
| Boys | 1.74 (1.41, 2.13)^***^ | 0.106 | 1.62 (1.29, 2.03)^***^ | 0.060 | 1.45 (1.01, 2.08)^*^ | 0.168 | 1.38 (0.92, 2.08) | 0.259 |
| Girls | 1.57 (1.32, 1.88)^***^ | 0.071 | 1.79 (1.44, 2.24)^***^ | 0.070 | 1.36 (1.06, 1.76)^*^ | 0.152 | 1.19 (0.90, 1.56) | 0.084 |
| Age stage | | | | | | | | |
| Early adolescence | 1.95 (1.41, 2.70)^***^ | 0.103 | 1.75 (1.27, 2.41)^***^ | 0.074 | 1.62 (0.89, 2.97) | 0.196 | 1.22 (0.62, 2.39) | 0.188 |
| Middle adolescence | 1.83 (1.49, 2.26)^***^ | 0.095 | 1.70 (1.43, 2.01)^***^ | 0.063 | 1.42 (1.06, 1.89)^*^ | 0.144 | 1.14 (0.85, 1.52) | 0.113 |
| Late adolescence | 1.50 (1.13, 1.99)^**^ | 0.078 | 1.62 (1.15, 2.27)^***^ | 0.047 | 1.02 (0.71, 1.46) | 0.196 | 1.49 (0.96, 2.23) | 0.165 |

**Notes:**

^a^ Adjusting for sociodemographic variables, and baseline status of elevated depressive symptoms.

^b^ Adjusting for sociodemographic variables, and baseline status of elevated generalized anxiety symptoms.

^*^p＜0.05, ^**^p＜0.01, ^***^p＜0.001.

T1: time 1; T2: time 2; T3: time 3.

IU: Intolerance of uncertainty; OR: adjusted odds ratio; CI: confidence interval.

**Table S5** Fit indices for the longitudinal measurement invariance of the key study variables.

| **Variables** | **Model test** | **χ2** | **df** | **CFI** | **TLI** | **RMSEA** | **SRMR** | **ΔCFI** | **ΔRMSEA** | **ΔSRMR** |
| --- | --- | --- | --- | --- | --- | --- | --- | --- | --- | --- |
| Generalized anxiety symptoms | Configural invariance | 1014.990^*^ | 165 | 0.983 | 0.978 | 0.031 | 0.015 | - | - | - |
|  | Weak invariance | 1050.323^*^ | 177 | 0.983 | 0.979 | 0.031 | 0.017 | 0 | 0 | 0.002 |
|  | **Strong invariance** | **1119.247**^*^ | **189** | **0.981** | **0.979** | **0.030** | **0.017** | **0.002** | **0.001** | **0** |
|  | Strict invariance | 1827.993^*^ | 203 | 0.968 | 0.966 | 0.039 | 0.033 | 0.013 | 0.009 | 0.016 |
| Depressive symptoms | Configural invariance | 2616.700^*^ | 294 | 0.961 | 0.953 | 0.039 | 0.026 | - | - | - |
|  | Weak invariance | 2662.930^*^ | 310 | 0.960 | 0.955 | 0.038 | 0.028 | 0.001 | 0.001 | 0.002 |
|  | **Strong invariance** | **2815.523**^*^ | **326** | **0.958** | **0.955** | **0.038** | **0.028** | **0.002** | **0** | **0** |
|  | Strict invariance | 3733.846^*^ | 344 | 0.942 | 0.941 | 0.043 | 0.044 | 0.016 | 0.005 | 0.016 |

**Note:**

Values in bold indicate established model of measurement invariance.

-: not available; df: degree of freedom; CFI: comparative fit index; RMSEA: root mean square error of approximation; SRMR: standardized root mean residual.
